# Best Practices from Eight European Dementia-Friendly Study Cases of Innovation

**DOI:** 10.3390/ijerph192114233

**Published:** 2022-10-31

**Authors:** Elisa Pozo Menéndez, Ester Higueras García

**Affiliations:** 1 Grupo ABIO, Universidad Politécnica de Madrid, 28040 Madrid, Spain; 2 Matia Research Institute, 28020 Madrid, Spain

**Keywords:** age-friendly communities, built environment, ageing in place, long-term care settings, quality of life

## Abstract

The concept of dementia-friendly cities and communities has achieved great dissemination in the international context since 2016. Although it is usually related with community networks and services, evidence and guidelines show the close relationship between the built environment design, health promotion, and the preservation of relationships with the local surroundings. Recent publications emphasize best practices in urban areas and care management. However, this is a very complex reality in each country depending on the sociosanitary services, the demographic, and geographic structure and many other different aspects including cultural ones. Moreover, design should also consider not only basic aspects such as habitability or universal accessibility, but also heritage, identity, and the feeling of normalized living. Knowledge about international experiences and innovative approaches is, as yet, an object of study as demographic ageing is still challenging all the welfare systems, especially in Europe. This study presents eight descriptive study cases in three different European countries—the United Kingdom, Belgium, and The Netherlands—to analyze the relationship between dementia-friendly initiatives and their intersection with design, urban planning and the provision of care. The results can provide strategic lines for development and innovation towards dementia-friendly societies and cities achieving SDG numbers 3 and 11 simultaneously.

## 1. Introduction

The concept of dementia-friendly cities and communities has achieved great dissemination in the international context since 2016 [1]. The ‘dementia friends’ initiative was first started in Japan in 2004, when the Japanese government announced the change of the word for ‘dementia’ which was associated with negative connotations to another more inclusive word (Japanese word for ‘dementia’, ‘Chiho’, was changed to ‘Ninchi-sho’, meaning cognitive disorder, by Japan’s government in 2004 [2]). From that moment, the national campaign ‘10-Year Plan to Understand Dementia and Build Community Networks’ was launched, which activated the creation of several local initiatives around Dementia-Friendly Communities, and later inspired the Dementia Friends program by the Alzheimer’s Society in the United Kingdom in 2013 [2]. The World Health Organization defines a dementia inclusive society as ‘one where people with dementia and their caregivers experience no stigma and fully enjoy independent living and quality of life’ [3], where accessibility is defined as the ability for older people, people with dementia, and people with disabilities to use public physical and social environments safely’ [4].

There are multiple projects and innovative practices that have adapted this general framework to their local context [5,6]. The main aim of dementia-friendly cities and communities is to improve the quality of life of people with dementia and their relatives through integration and inclusion of people with dementia in society and community life. The objectives are to reduce the stigma about dementia and increase social awareness about this condition, as well as to empower people with dementia to promote their autonomy and capacities to maximize their control over their own lives [1]. According to the Alzheimer’s Society definition ‘Dementia-friendly communities are vital in helping people live well with dementia and remain a part of their community’ [2].

Even though these kinds of initiatives have been normally focused on social and community aspects, in 2020 Alzheimer’s Disease International published the ‘World Alzheimer Report. Design, dignity, dementia: dementia-related design and the built environment’ [7], where the second volume included the analysis of 85 architectural projects and inspiring study cases at the international level that were selected from an international survey and a self-reported questionnaire. From this list, best-practices offer a variety of projects including elderly day care centers (*n* = 21, 25%), care homes (*n* = 58, 68%), public equipment (*n* = 5, 0.06%), and hospitals (*n* = 1, 0.01%); whereas almost a third of there (*n* = 25, 29%) are European cases. From this selection, there are also examples of elderly day care centers (*n* = 3, 19% from the total of elderly day care centers), care homes (*n* = 18, 31% from the total of care homes), public equipment (*n* = 2, 40% from the total of public equipment), and hospitals (*n* = 1, 100% from the total of hospitals). Many of these projects are in the United Kingdom (10) and Ireland (2), being representative of 48% of the best practices that were identified in Europe (Table 1).

Considering the different care models across regions and countries in Europe, the main objective of this research is to gather information about projects in Europe that have been recognized as best practice, in order to analyze and evaluate multiple variables considering both the community support and the role of the built environment in each local project. The physical environment of proximity has a major impact for older people’s lives regarding their quality of life as it influences the variety of activities that can be done, as well as socializing [8]. Residential habitat is a determinant in the ageing process, not only the housing, but also the other alternatives where the person lives when high dependency appears, and extra care and support are needed.

**Figure 1:**
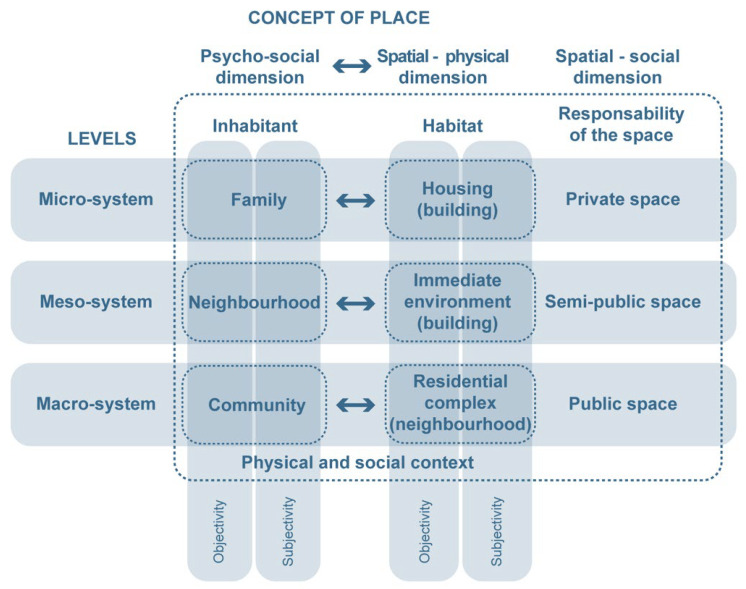
Connection between the psycho-social, spatial, and physical dimensions that configure the concept of a place. Source: Own elaboration, adapted from Haramoto, in Bernales (2021).

Moreover, according to a transactional perspective, people (psychological processes) and their environments are considered as an inseparable unit that are completed by temporal qualities to define events as a holistic unity [9,10]. In this sense, routines in daily life need to consider the multiple levels that are established by Haramoto (Figure 1). This interpretation is perfectly aligned with Lynch’s theory of the image of the city, in which the images that are created by the individual and the group can be coincident, and so, the legibility of a given place is a key aspect of its identity and it can be defined by five distinctive elements (edge, path, node, landmark, and district) [11].

In this context, the focus of this study is put in the urban design, the street layout, and urban planning, as guidelines and recommendations to highlight the importance of the urban environment to promote dementia-friendliness within a community [12,13], as well as health promotion in cities [14,15,16,17].

## 2. Materials and Methods

### 2.1. Selection of Study Cases

This research is based on a descriptive case study in order to detail the particularities of a specific phenomenon in different scenarios of the real world [18,19]. The methodology consists of a deep analysis of one or several cases considering multiple techniques in order to collect multiple data that answer a specific objective [20].

The European best-practice study cases have been selected from the following databases as the most relevant sources for dementia and Alzheimer information and resources:

Alzheimer Disease International [21] *Dementia-friendly communities. Global developments*.

Alzheimer’s Society [22]. *List of Dementia Friendly Communities.*

*Dementia-friendly community case studies across Europe. A mapping commissioned by the European Foundations’ Initiative on Dementia* [23].

The input keywords were ‘dementia-friendly communities’ or/and ‘dementia-friendly design’.

The selection criteria were to have an award or prize or publication. Initially, the search was oriented to identify regulations or guidelines about urban environments that consider the specific necessities of people with dementia. However, after the first review of the project list of these databases, the results showed projects based on care homes and dementia-friendly initiatives. Thus, the selection criteria were amplified to learn from these experiences and their relationship with public space and urban design (Table 2).

### 2.2. Visits and Data Collection

During the research, the systematic data collection was structured in the following steps:

1. Review of existing guidelines, national, and local plans that promote ‘dementia-friendly communities and cities’ or ‘dementia-friendly’ initiatives’.
2. Guided visits to the building or project. In the case of buildings, analysis and observation of the urban environment and outdoor spaces were undertaken. The observation technique consisted of ‘procedures and instruments that are used by the researcher in order to experience social realities, as well as seeing the people in their usual context where they usually develop their routines, including the social, cultural, and physical environment’ [24]. All the visits included a non-participatory observation that consisted of a guided visit with complementary information about their functioning and programs. Pictures were taken under the consent of the responsible person, otherwise public media were used.
3. Cartographic drawings of each study case that were based on the key elements of Lynch were developed in order to understand the different urban contexts regarding both positive and negative aspects of building a dementia-friendly environment and facility.
4. Semi-structured interviews with the responsible person (before and after the visit). According to Zarco et al. (2019) [24], interviews have the main objective of ‘establishing a dialogue that enables obtaining deeper information about the contexts’. Although the content of the interviews was designed following common criteria and structure, the different typologies and contexts of each project made it necessary to adapt it to each specific case, in order to consider the particular aspects of their best practice.
5. The information was systematically analyzed in different categories to describe and identify the main elements that have been considered to create dementia-friendly environments.
6. Finally, secondary resources were consulted in order to complete some missing information.

### 2.3. Interviews

The interviews took place on the same day as the project visit, most of the time with visual access to common spaces. The interlocutors were the directors of the project or the person in charge. The average length of the interviews was 71 minutes in total, with high variability depending on the project typology. The content of the semi-structured interviews included the following items (Table 3).

A total of 8 interviews were registered. The data were transcribed in English and analyzed. Due to the multiple lengths, content, and scripts, the analysis of the information was based on the categories and elements analysis in relationship to the different aspects of the design (Table 4).

## 3. Results

The eight projects are described separately in detail in this section, including the information from interviews that were most relevant regarding specific aspects of the work.

### 3.1. United Kingdom

The Anglo-Saxon care model is applied in Great Britain and Ireland, and it is based on the private initiative, considering social assistance as the last resort, relying on the market to regulate offers and services. The role of the state is above all to intervene in those points where the market does not reach or satisfy the needs. A total of three study cases were selected because of their innovative character.

#### 3.1.1. Discovery Museum, Newcastle-upon-Tyne

**Figure 2:**
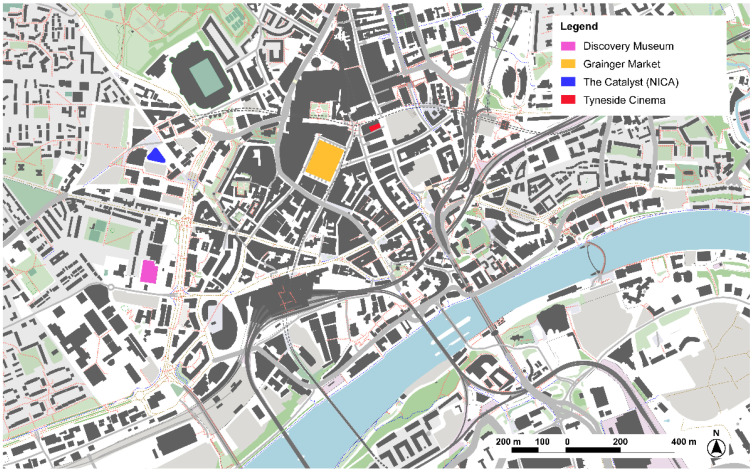
Main innovative dementia-friendly projects in Newcastle-upon-Tyne, United Kingdom. Source: Own elaboration.

Newcastle-upon-Tyne is located in the northeast of England. It has a population of 279,100 inhabitants and a density of 2469.9 hab/km^2^. Recently, in 2020, the National Innovation Centre for Ageing has been inaugurated, positioning the city at the leading edge on innovation and research on ageing. In this context, the Slow Shopping and the Discovery Museum was selected as they work on the daily spaces that support autonomy for people with dementia: shops and local equipment (Figure 2). Slow Shopping (Slow shopping. Available at: https://www.slowshopping.org.uk/, accessed on 10 October 2022) is a non-profit organization to adapt local supermarkets to people with disabilities, specially focusing on people with dementia. It consists of a training program to make shops and supermarkets friendlier to people with dementia through special schedules for a quieter environment and extra support to enable people with dementia to continue to go about their usual routines. The same principle was also introduced in the Tyne and Wear Archives and Museums, a metropolitan network of museums and galleries. In this sense, from Slow Shopping the concept of Slow Museums has been developed, providing a specific schedule for people with dementia, where the visit is adapted and guided specifically for people with dementia and the tour guide is trained in order to manage conversations to engage participants.

‘*We have trained the staff up to be dementia-friends, so they’ve just got that little bit extra knowledge. So, if people with dementia are coming into the museum, the staff understand a little bit more and hopefully have a little bit more confidence to deal with anyone that needed help*’.

In addition, another program called *Platinum Programme* is also complementary to the Slow Museum initiative and it is offered for seniors above 50 years old. Reminiscence boxes are taken into different day care centers or nursing homes, so people become more familiar with the museum’s team and would feel comfortable to visit the museum more often.

‘*I would say I’ve got a lot of relationships with people like dementia care in Newcastle and Age UK. And they’re the ones who bring groups to me, but I also go and see them, so because I’ve got these good relationships with the members of staff, I’ll see them once a month. So, I think next week dementia care is coming in again. And I’ll take them around to the gallery or I’ll sit with some of the objects and things and Age UK, we’ve got… it’s a group called The Time Travellers. They called themselves The Time Travellers because they didn’t want to be known as the Age UK dementia group (…) we look at different periods of time and so that’s again once a month and they will meet in the museum with myself and I’ll do some sort of topic (…) that used to be once a week and so, I used to see this group once a week, which was great. The group got to know each other, they’d be seeing each other outside of the group, there were all people with early-stage dementia and their caregivers and so, they were making friends and I was seeing them like once a week*’.

Economic sustainability to support these kinds of programs is necessary, but when a natural bond is made, the activity remains active with a social engagement ‘*it just continues because it works well for both of us*’.

It is also interesting to highlight the social prescription that doctors might first recommend in case they see the possibility to make the person enjoy social life, as the network of museums are included in the list of recommendations ‘*What the doctors will say is* “*here’s a leaflet showing you what groups you can join at the museums of the art galleries (…) Why don’t you go along to this on a Wednesday afternoon and meet some new people? (…) You know, the Hancock Museum down the road is doing a group session to do with the Romans for a few weeks, why don’t you go along?*”’.

**Figure 3:**
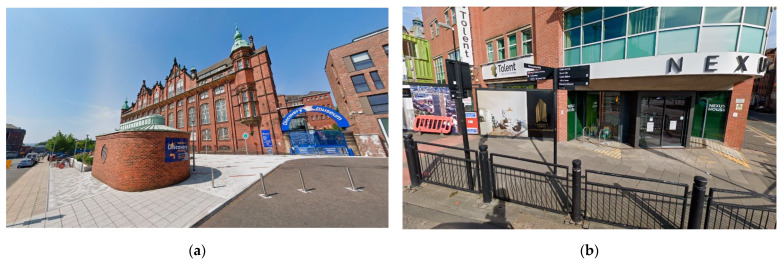
Elements of the built environment in Newcastle-upon-Tyne. Source: Google Street View, 2021. (**a**) The Discovery Museum is a museum from the Tyne and Wear Archives and Museums network. It holds a historic and scientific program, set in an ancient building from the XIX century from the Cooperative Wholesale Society. The main access has been refurbished creating a pedestrian entry. In the interior, the building is adapted according to universal accessibility criteria. (**b**) Across Newcastle’s streets it is usually easy to find signposts which indicate directions to the main buildings of the city. Also, informative panels can be found to better help wayfinding in the city.

Regarding the physical environment, the museums are accessible for physical impairment (ramps, toilets, wheelchair access, etc.), but they have also got to be adapted for older people and people with dementia: ‘*it’ll be less noisy, we’ll turn things down, there’ll be more seats out (…) a bit more signage as well if we’re doing slow museums, we can just make things a little more obvious…*’ (Figure 3).

The interview completed the information about the high level of awareness in society about ageing and dementia-friendly programs. Both formal and informal networks are promoted by different stakeholders, which enable more innovative projects for social inclusion in normalized environments, such as museums, local supermarkets, churches, cinemas, or municipal markets.

**Figure 4:**
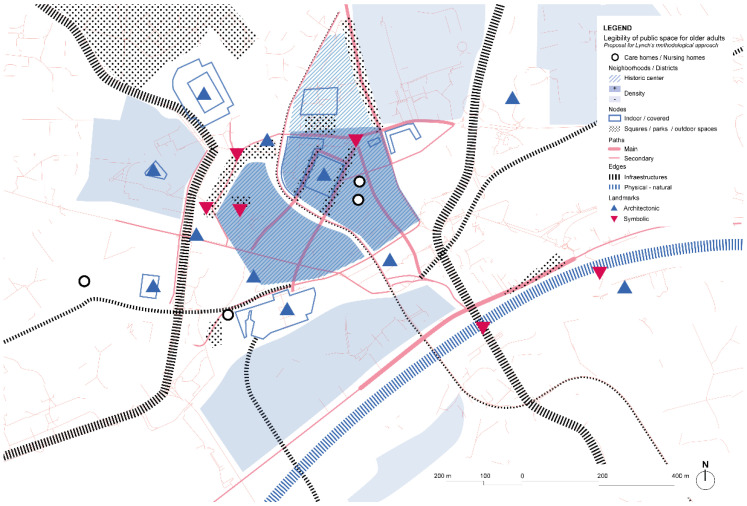
Newcastle city-center’s scheme based on Lynch’s theory. Source: Own elaboration.

Analysis of the city based on Lynch’s theory shows a great presence of infrastructure barriers that might be dangerous in terms of providing safety paths to walk along (Figure 4). Cul-de-sacs are usually found in some streets, although the main ones are easy to find and get oriented in thanks to clear visuals to different landmarks for both monuments and greater buildings that provide helpful information when visiting the city. Getting to the Discovery Museum might not be evident for some people that would want to join the program, whereas the Grainger market or the Tyneside Cinema in the city center are easily found in the city center and are well connected with pedestrian paths and public outdoor and indoor spaces. A lack of environmental wayfinding is compensated by signposts and maps in the streets.

#### 3.1.2. Harmonia Village, Dementia Village, CASCADE Project, Dover

**Figure 5:**
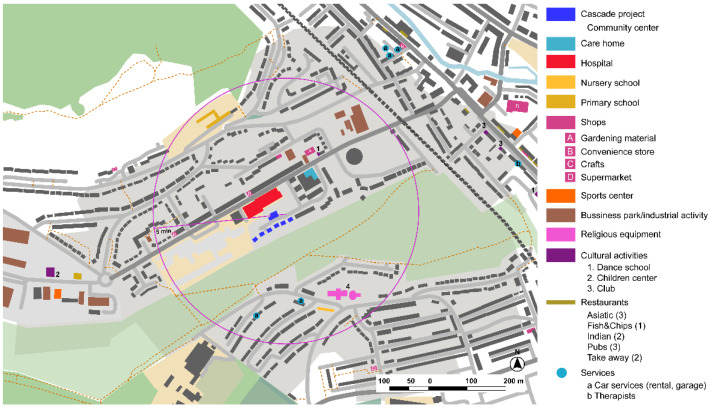
New Harmonia Village under CASCADE project and near surroundings, Dover, United Kingdom. Source: Own elaboration.

**Figure 6:**
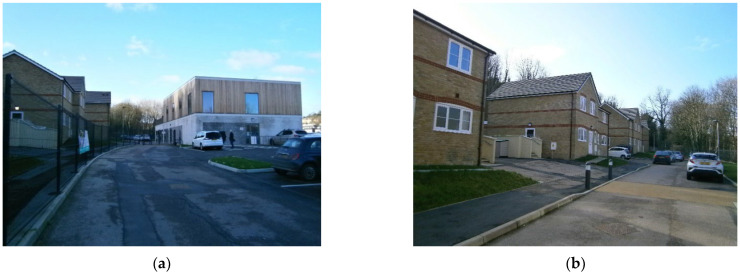
Selection of main pictures of the Harmonia Village project. Source: Own elaboration, 2020. (**a**) The complex is in a cul-de-sac street, just beyond several similar houses and near the Buckland Hospital. The main gate is through a metallic fence for greater security, preventing the persons that live there from going out and controlling the access for visitors. Just after the main gate, the community block welcomes visitors, families, and staff members. There is also a café and several spaces for workshops and community activities. (**b**) The care home is structured as six houses and an extra block for common services. Each one has five rooms, a living room, and a kitchen. The whole complex has been refurbished with a lift in each house and all the requirements to adapt it to accessibility standards.

Harmonia Village is the first ‘dementia village’ project in United Kingdom [25]. It consists of an urban renovation project where several abandoned houses have been refurbished to create a new care home model for people with dementia. The project is part of a greater consortium with multidisciplinary stakeholders within an international program called the Interreg 2 Seas Program [26], including another new facility for people with dementia in the UK (Figure 5 and Figure 6).

The project is articulated under five key principles: to emphasize the individual capabilities and desires of the person, to assure a holistic and integrated care, to create opportunities to develop a meaningful life, to promote a positive vision of dementia in the community and in society, and to create a safe environment within the community for an independent life.

‘*We have been looking for other facilities, we know the necessities for the residents very well. We know the case of De Hogeweyk in Holland for long time, but it’s very expensive. (…) We could not replicate all the services in the community inside the walls (…) And we felt that was quite isolating. We want people to use the community facilities and become more normal for people with dementia to be in the community*’.

One of the main objectives of the project is to create a new care home model for people with dementia, including sensors for monitoring and support in their daily life. The aim is to keep the person integrated in their community and at the same time to maintain their quality of life and well-being.

‘*It’s only when they deviate from the norm when you go and have a look at what’s happened. So, once we’ve got that profile for each person the system will monitor them on an ongoing basis and then on a weekly or monthly basis… and the clinical staff will look at the information and they’ll see what sort of correlations there are and trends*’.

‘*One of the things we’re measuring here is the economic impact of this facility on the local community. So, the construction contract was only open to local companies. We’re basically employing local people and all the technology, everything we’re using is locally sourced (…) instead of having a hairdresser’s in here, or a cinema…we’re going to go out and use those services in the local community. And thereby have a beneficial economic impact also*’.

**Figure 7:**
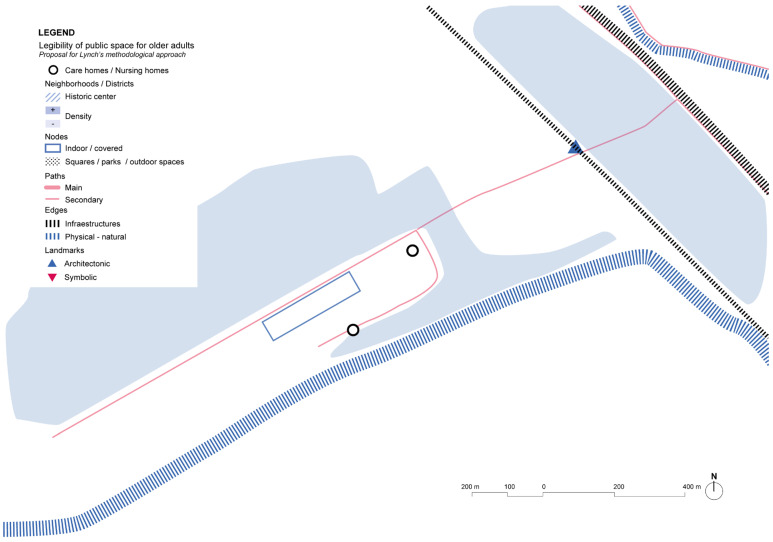
Harmonia Village’s surroundings’ scheme based on Lynch’s theory. Source: Own elaboration.

The analysis of the site where Harmonia Village is located based on Lynch’s elements shows a lack of diversity of elements (Figure 7). The place where it is located is a cul-de-sac street that is surrounded by green hills. On the other hand, connectivity with the rest of the town is not evident, although it cannot be mistaken as there is only one main road, while pedestrian accessibility is not assured as infrastructure barriers separate the site from the rest of the town. The maintenance of the image of the normal dwellings is an advantage in terms of creating a familiar environment and optimizing resources, creating a new service hub in this area. However, it is difficult to find a diversity of other activities or services around this area of the city.

#### 3.1.3. Harmony House, Respite Home, CASCADE Project, Rochester

**Figure 8:**
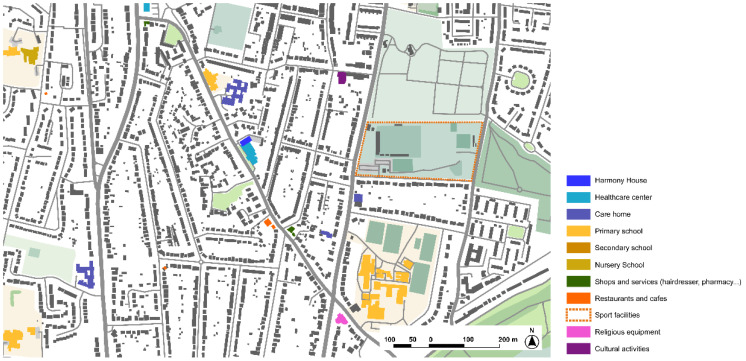
Site of the Harmony House under CASCADE project in Rochester, United Kingdom. Source: Own elaboration.

Harmony House is a new respite center in Rochester (2019) for short-term stays for people with dementia and their carers or relatives. The center is adjacent to a pre-existing sociosanitary center and integrated in the community (Figure 8), as the main purpose and objective of the project is to promote autonomy and normalized living for people with dementia.

In addition, taking advantage of local resources is also a main aim of the project, involving different stakeholders and creating synergic networks.

‘*One of the aspects that we wanted to look at (…) was focusing on healthcare professionals (…) care professionals, such as the voluntary sector colleagues, the charity sector, the local authority (…), and then, our European colleagues had brought in the aspect of tourism, which was something we’d not thought of at all. However here in Rochester we’re in a really good spot for tourism. We have Dickens (…), there’s a castle, there are museums, we have the dockyard down the road, we have access to other places such as Leeds castle (…) just because somebody’s got dementia doesn’t mean to say they can’t ever go on holiday again*’.

**Figure 9:**
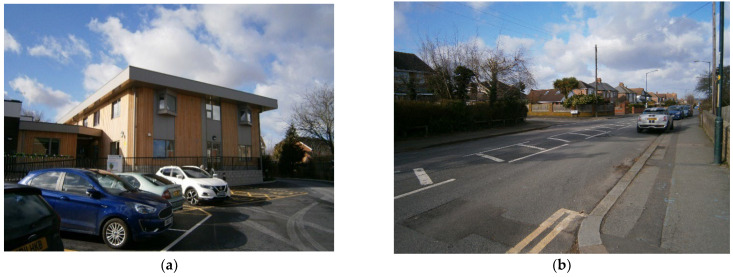
Selection of main pictures of the Harmonia Village project. Source: Own elaboration, 2020. (**a**) The center is in a residential area. Although private vehicles dominate the outdoor spaces, (**b**) Harmonia House is near several local services that encourages interactions with the surroundings.

The project needs to evaluate both the social and economic impact in the local context as well as the use of new technologies to promote autonomy and freedom of movement for people with dementia and the first symptoms of wandering. On the other hand, the inclusion process and dementia-friendliness are, as yet, tough both in social and physical environments (Figure 9):

‘*As well, we did have some pushback from the local residents, we had a couple of local residents’ meetings, we invited them to look at plans (…) but people didn’t understand what we were trying to do and came with preconceptions of what they thought dementia was*’.

One of the most interesting aspects is the team process towards a dementia-friendly approach and the empowerment of the community to support people with dementia through specific training, empathy, and teamwork around all the individual issues that arise from the care process in a continuous learning path, both in the team and out in the relationship with the community.

‘*She’s not a nurse, has no nursing experience at all (…) what we wanted was somebody that shared our enthusiasm for the project (…) with the whole of the team we recruited for values more than skills. (…) We have two senior personal assistants, and we have seven personal assistants and a number of those have not come from care, which is great I think… (…) because we’re not about caring we want to be about enabling*’.

Finally, an intersection with the design of public spaces should also be combined with normalization of living environments assuring accessibility above all.

‘*Sometimes we really focus on risk too much. So, in terms of public spaces (…) we’ve got some really nice public spaces in Medway, some really nice garden areas, but a lot of them have little gates on them and for somebody living with dementia that is a nightmare. It’s bad enough if you haven’t got dementia and you don’t know how to get in (…) the accessibility in terms of being able to actually enter that space in the first place is really difficult and quite challenging*’.

**Figure 10:**
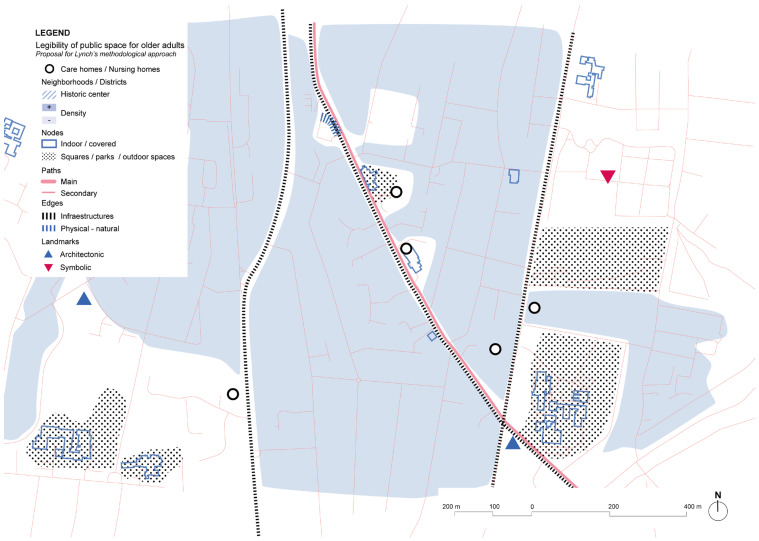
Harmony House and its immediate surrounding’s scheme based on Lynch’s theory. Source: Own elaboration.

Analysis of the urban environment around Harmonia House shows a low-density neighborhood with a lack of landmarks that might contribute to support wayfinding (Figure 10). There are some services that are close to the main street, as well as many other major facilities, mainly educational centers, but pedestrian accessibility and friendlier environments are a major aspect that are yet to be achieved in the general layout of the streets of this area of the city.

### 3.2. Belgium

The Belgian model of care is the so-called ‘continental’ model, similar to the Nordic one, where the State assumes a great part of the services and support, but there is also a major presence of private assurances. The three study cases were selected from the main repositories of best-practice in dementia-friendly communities.

#### 3.2.1. Woonzorgcentrum (WZC) De Weister, Nursing Home, Aalbeke, Kortrijk

**Figure 11:**
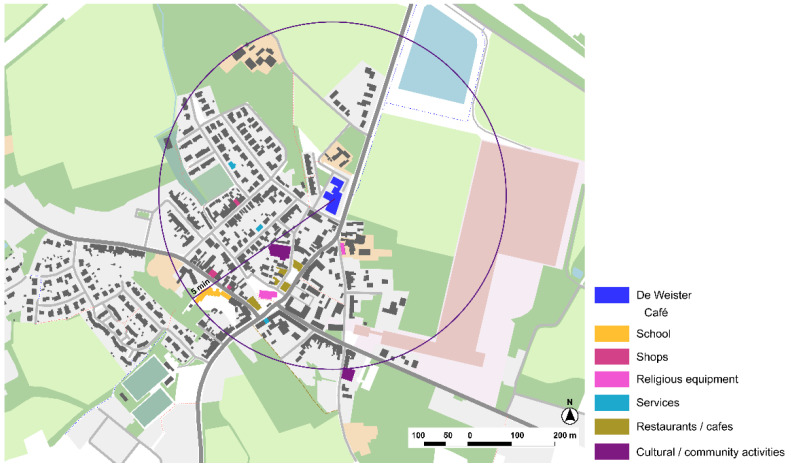
De Weister’s location in Aalbeke, Kortrijk, Belgium. Source: Own elaboration.

The Woonzorgcentrum (WZC) De Weister is a nursing home in Kortrijk for people with dementia and high dependency. It opened in 2012 and was founded by Zorg Kortrijk, the Public Center for Social Welfare (Figure 11). The city of Kortrijk, as many other cities in Flanders (Belgium), has been working towards dementia-friendliness in a structured plan. The Flemish Government established the first Dementia Plan in 2010, which is now in its second version.

Additionally, cities can also develop a dementia-plan, which is the case of Kortrijk.

‘*It’s all about stigma, acceptance… and if you can create this, then you have a dementia-friendly community (…) So, the first thing is that we have to create citizenship, so it is about making people part of a community (…); the second thing is getting rid of the stigma. And then a healthy mind in a healthy body (…) so, health promotion*’.

The center is structured in three convivial units, with 14–16 rooms following the principles of small-scale design. Each unit is articulated around a main living room, with an integrated accessible kitchen, where the residents are encouraged to join the daily routines.

‘*We create a social context so people can meet and that’s in our living rooms (…) we really live in the living room with the kitchen, we cook together with the residents, so we create a relational context or a social context where people can meet. It’s not because you’re in a nursing home you’re staying in your room. We live together, okay? But there’s free choice*’.

**Figure 12:**
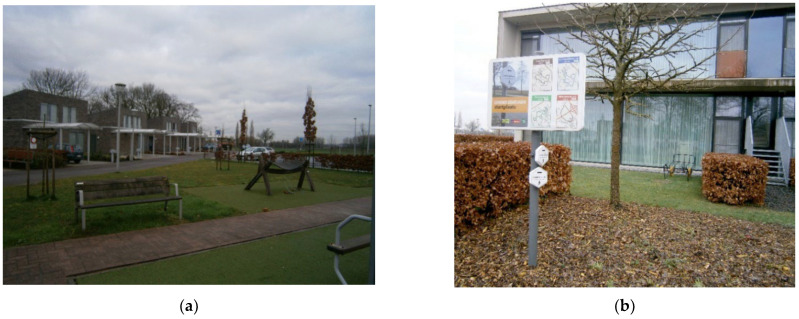
Selection of main pictures of the WZC De Weister project. Source: Own elaboration, 2020. (**a**) Near the WZC De Weister, there is an intergenerational park with musical instruments that stimulate social interaction. The park also includes gymnastic elements to train and keep fit with low impact exercises. (**b**) An innovative project that answers all the strategic lines that are defined in the dementia-friendly plan is the *Reminiscence Walks,* where four itineraries have been defined with the local community, connecting heritage elements across the village and promoting healthy habits and social interactions.

**Figure 13:**
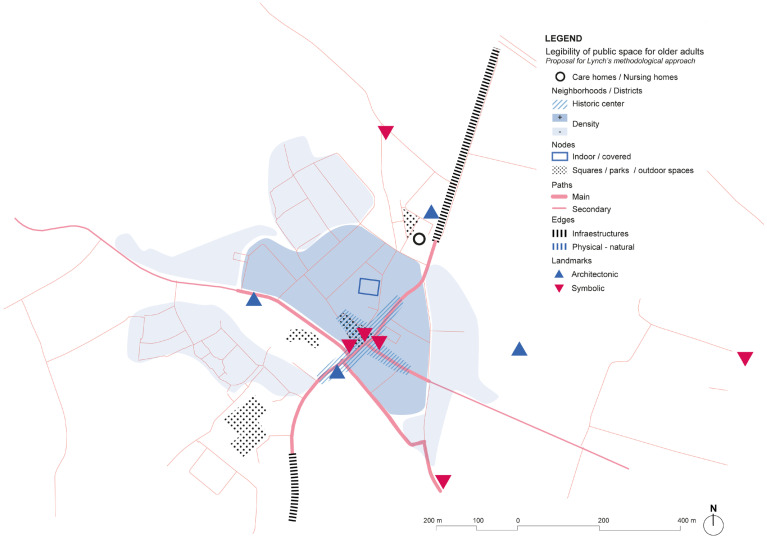
De Weister center and its surroundings’ scheme based on Lynch’s theory. Source: Own elaboration.

The most important thing is the connectivity and integration with the local community as part of the daily routines of the center. Families have an active role in decision-making and planning activities, as well as community, in terms of informal care givers, volunteers, or associations that ‘dynamize’ or organize different activities. This active interaction with the community shapes the architectonic project (Figure 12). The continuity with urban spaces is important in the immediate surroundings, with a green area, a park, and public equipment both for children and people with dementia (Figure 13).

‘*… the intergenerational move garden (…) the aim was to make people move, improve intergenerational contacts through movement, but also through music. Because music is also very important for people with dementia. (…) we worked also together with the local community, and we chose those things to move (…)*’.

#### 3.2.2. Foton, Expertise Center of Dementia of Flanders, Bruges

**Figure 14:**
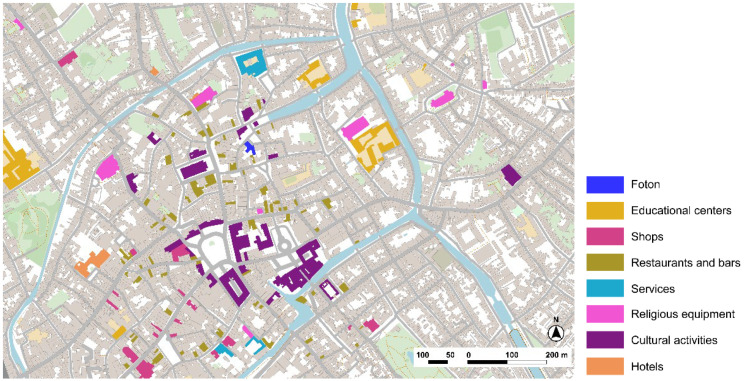
Foton Expertise Center of Dementia and city center of Bruges, Belgium. Source: Own elaboration.

Foton is an expert center for dementia in Bruges, Flanders, an initiative from the non-profit organization Familiezorg West-Vlaanderen inspired by the Alzheimer Scotland’s publication in 2001, ‘Creating dementia friendly communities: a guide’ (Figure 14). In 2010, Foton started to develop the initiative *Together for a dementia-friendly Bruges!* The main purpose was to improve the quality of life of people with dementia in their local environment. Foton is a reference center for assessment about dementia care and family support, but it also creates and promotes innovative ways to engage local communities towards dementia-friendly initiatives.

Engaging with local shops and pharmacists was one of the long-term projects under a 5-year strategic plan that has been developed in order to raise awareness about dementia, as well as to contribute to reduce the stigma about it. The establishments would put the red knot logo on their showcase and receive specific training to be more confident to interact with people with dementia.

‘*We have made a movie, where some of the persons that come here acted to make a movie to show to the other people who are working in the shops how you can communicate with people with dementia. (…) So, they had the opportunity to have a few evenings that they can talk about it. So, for the whole city, it was a little bit much. So, the group of volunteers went to a few streets and they visited the shop owners or the people who work in the shop to talk about the initiative, to give a sticker. So some people were ready to put it on the window, other people say ‘oh, we will do it’. And they don’t do it (…). That’s why at one shop you can see them, at the other not… (…) But there has also have been a campaign for the pharmacist. (…) So, one day they distribute the information (…) there are a lot of people of the of the area who come to the pharmacy…*’.

**Figure 15:**
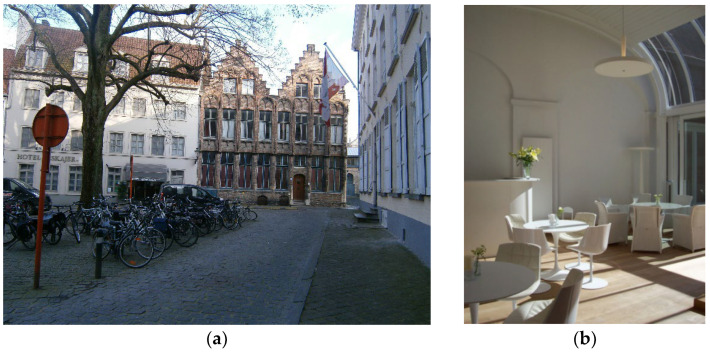
Selection of main pictures of the Fotonhouse in Bruges. Source: (**a**) Foton, (**b**) Own elaboration, 2020. (**a**) The Fotonhouse is in the city center of Bruges, integrated in the urban tissue and with connectivity to open public spaces in a quiet and calm environment. (**b**) The Kopje Troost room in the Fotonhouse has offered a quiet place since 2005 for social meetings and both formal and informal meetings between people with dementia, families, and caregivers. They offer a community library with different audio-visual material, cultural and musical activities, memory choirs that are run by volunteers, and many other celebrations over the year.

Also, the Fotonhouse offers a quiet space, the Kopje Troost, which is open to anybody in the community and where people can join and meet (Figure 15):

‘*You cannot call it café, but it’s a quiet place, where you can drink something, where you can have a chat also, what are lonely people, most of them are people who don’t have too much family, or they have a disease… or some little problem. They come here and they can have a cup of coffee or tea*’.

**Figure 16:**
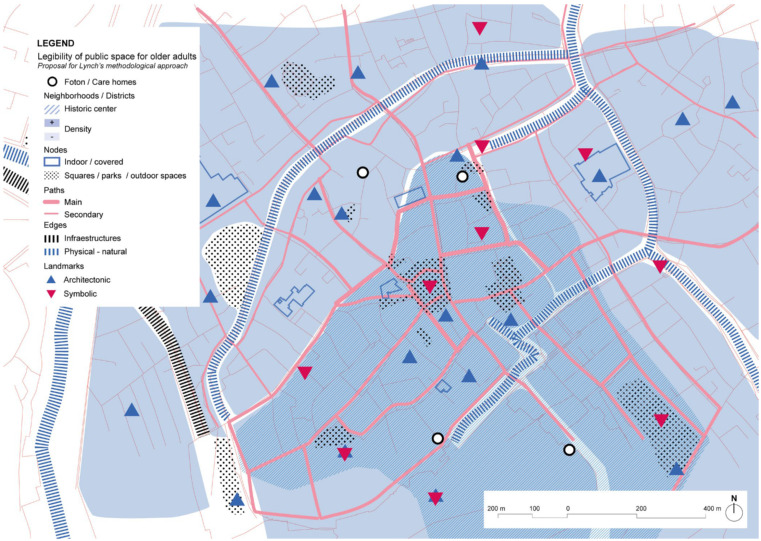
Foton center in the city of Bruges and its surroundings’ scheme based on Lynch’s theory. Source: Own elaboration.

Analysis of the city center of Bruges, where Foton is located shows a rich diversity of services, shops, and commerce. However, part of the offer is dedicated to tourism especially during summertime, but still, it is possible to see the mixture of activities in the urban tissue (Figure 16). Considering Lynch’s elements, the number of landmarks is important in the urban streetscape, making it easy to navigate around the city looking for the reference towers of higher buildings than appear in the low-rise landscape. The absence of infrastructure that might create barriers is also significant, as the main ones are the channels, but the multiples bridges solve connectivity between areas.

#### 3.2.3. Huis Perrekes, Osterloo, Geel

**Figure 17:**
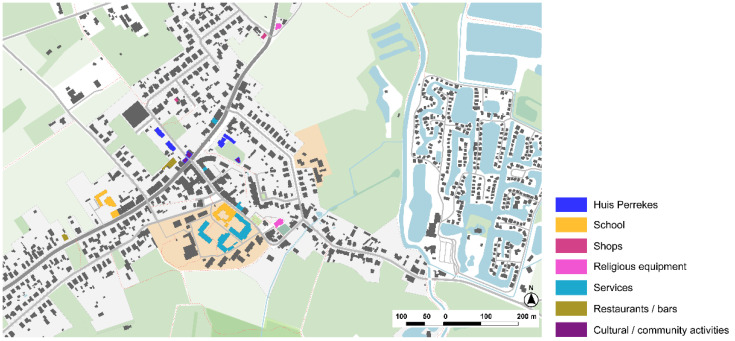
Main buildings of Huis Perrekes and services in Osterloo, Geel, Belgium. Source: Own elaboration.

Huis Perrekes is a unique project in the articulation of care strategies within a small-scale project in the village of Osterloo. Huis Perrekes was first created in 1986 and it consisted of four homes that were integrated in the urban tissue (Figure 17). In 2003, a social study was developed in the community with the purpose of identifing the strategic lines in the medium- and long-term, both of Huis Perrekes and the community. Each Huis Perrekes house offers a normalized routine, people can engage with daily activities and participate in domestic life, such as preparing breakfast, making bread at home, cooking, cleaning, gardening, or growing vegetables.

‘*… All the people that live here or stay here for short stay… we know the whole continuum of the possibilities they all have after a dementia diagnosis (…). In this group, it’s very heterogeneous so there is a very big mix of types of dementia stages, so it’s not only for older people with dementia, but also for young people with dementia. It’s a mix, we don’t have a separate group because we think every person with dementia needs a care (…) which is individually adapted and (…) no matter the age. So, in every house live about 15 or 16 people with dementia and every group is heterogeneous. It’s not that people have to move from the house when they develop further in their process. (…) It’s a choice to do. It’s like this because then people can manage and mean more for each other…*’.

**Figure 18:**
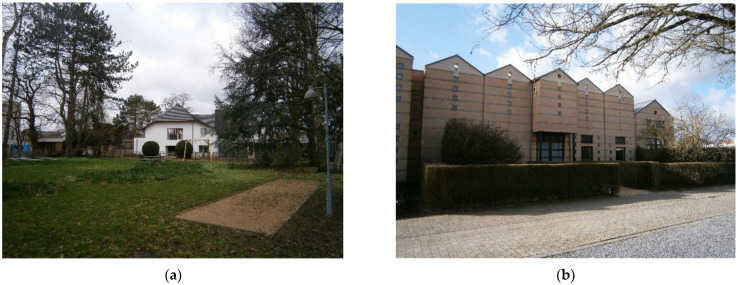
Main buildings of Huis Perrekes in Osterloo. Source: Own elaboration, 2020. (**a**) The backyard of the Huis Perrekes’ villa is a garden with different amenities for intergenerational use, such as swings or a petanque court. The garden offers a quiet place to walk around or stay, connected both with the indoor spaces of the villa as well as to the outdoor spaces of the village. (**b**) Huis Perrekes’ houses are completely integrated in their surroundings. The small-scale design enables normalized living and continuity within the community and urban landscape. The public space is pedestrian and bike-friendly, with slow streets and quiet areas.

Also, community spaces are open to other kinds of workshops and activities such as knitting, lectures, festivals, choirs, concerts, or farming that are usually developed with local stakeholders and partners (Figure 18). Heterogeneity of cultural and artistic activities offer multiple opportunities to join and connect in the community and remain active and part of it.

‘*We try to reach out by doing things that we do in the Huis Perrekes like the choir, all things that we do with textiles, but also (…) we organize readings or organize exhibitions, a lot of things where we invite families and people from the neighborhoods and that’s also why the garden is open, the gate is always unlocked, not during the night. So people who work or walk or bike in the neighborhood, they can come in, and they walk here and take a rest. The toilets of the building, they are also always open, so people always can… or, for example in summer, we had a freezer in the garden house (…) we put a board outside you can buy glace here and then people could walk by come in take a glace…*’.

**Figure 19:**
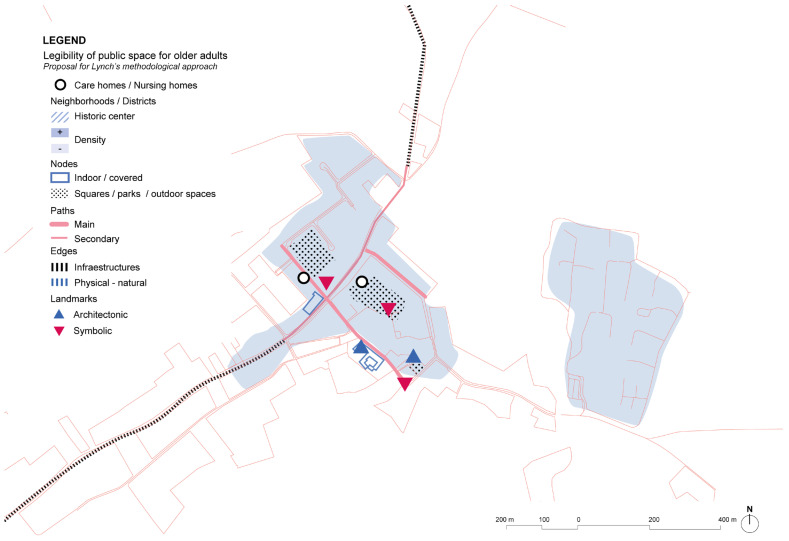
Huis Perrekes center in the town of Osterloo and its surroundings’ scheme based on Lynch’s theory. Source: Own elaboration.

The analysis of Osterloo shows a small town that is connected by a main road, but it is paved and treated as a pedestrian area in the city center, so the effect of barriers is not present (Figure 19). There are only main services in the town, but cultural activities are provided by Huis Perrekes being an intergenerational community resource. Considering Lynch’s elements, landmarks are not very prominent, but the small size of the town and the clear structure of the roads and paths makes it easy to navigate in a familiar and safe surrounding.

### 3.3. The Netherlands

The Netherlands can be considered as a Nordic model, where the state assumes a big role in the social and sanitary services. The aim is to remain at home, and projects such as the Buurtzorg are being promoted with high impact, which consists of nursing cooperatives that work at the neighborhood scale to provide personalized care at home. Focusing on the built environment, two projects have been selected.

#### 3.3.1. De Hogeweyk, Dementia Village, Weesp

**Figure 20:**
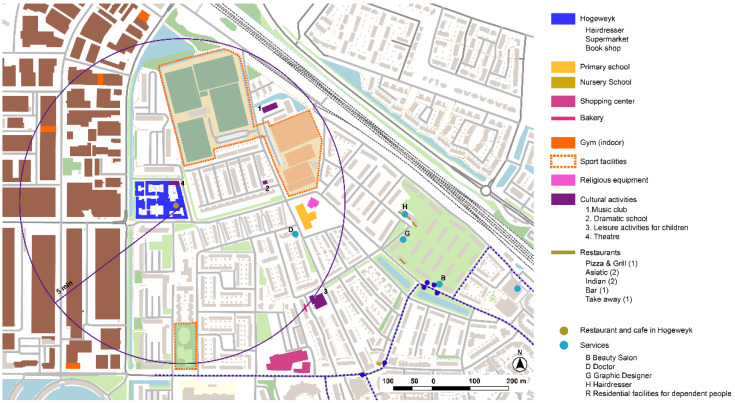
Analysis of the close surroundings of the De Hogeweyk project in Weesp. Source: Own elaboration.

De Hogeweyk is an innovative project that is commonly known as ‘dementia village’ that came together in 2002 after a long process of transformation into a normalized living for people with dementia (Figure 20). The design of the center recalls usual spaces in an urban area in The Netherlands, recreating equipment and amenities from normalized spaces, such as green spaces, public squares, a restaurant, shops, or a supermarket. Behind a normalized design, the different services of a care home are distributed in the center, which is also divided into small houses.

‘*…Everybody was living together towards a small group with like-minded people, so we developed the lifestyles, so we matched people in a group of 11 people where we brought back the normal daily living activities (…) We made a supermarket in that old building so you could go to the supermarket, we created club rooms and an event office outside the wards, so you have to go to your club event (…) You make your individual choice, you go to a club, to a place where you meet other people, where you do nice things you like. It’s your personal choice to go over there (…). We moved from 3 groups of 11 with wards, towards a house for 6, 7, or 8 people. You have immediately the quietness of a normal house where you have your own front door, where you go inside and nobody else comes there, unless you have to do something there (…) Wards give a lot of rumor noises, you don’t recognize people (…) You reduce the stress of 11 to 8 (…) And you reduce a lot of the stress and agitation because it’s a home, it’s a real house instead of a living room with wards. And a front door you can step outside, and you really walk outside, and it reduces also stress*’.

**Figure 21:**
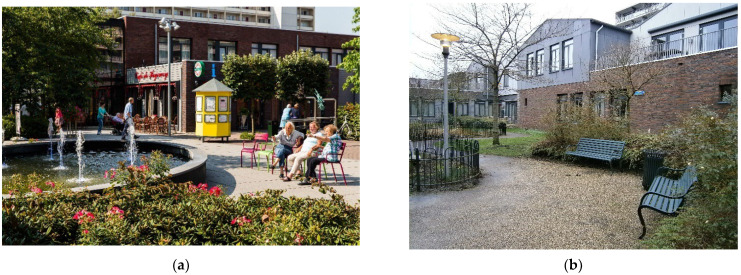
Outdoor spaces in De Hogeweyk. Source: (**a**) De Hogeweyk, (**b**) Own elaboration, 2020. (**a**) The public space at the entrance of De Hogeweyk is a public square with different stay areas with amenities such as a pond and fountain, a big chess board, terraces, and different spots to stop by and rest or gather. Natural stimuli such as birds or ducks are part of the biodiversity in the outdoor space. (**b**) A total of 23 houses are organized in two-story buildings around outdoor gardens with different identities and designs. There are benches, lamps, and fences as if it was any other quiet public space in the city.

The De Hogeweyk model acknowledges the importance of the built environment for people living with dementia (Figure 21). After 20 years of work, they now foresee interesting work towards creating friendlier surrounding environments and creating more interactions and synergies within the local context.

‘*So we must go back to what human beings really want and need and then apply the right support for the disease and those outcomes (…) if you compare the place at home—because the policy in The Netherlands is stay at home as long as possible—with good support, health support, home care support, etc… but in the end, for a person living with dementia that apartment or that house is often a cage, it’s closed, and the relative wants to keep mum or dad inside because outside it’s unsafe. And mum will walk away and so, mum is locked up (…) so, mum becomes aggressive or is lying (…) And here you get more freedom and you can you get 24 × 7 professional support so you see a different mum often here (…) Challenging behavior—behavior we don’t understand—is still part of what we have to deal with, we still have a locked door over there which I don’t like, so I hope with the new law implemented in The Netherlands we can unlock the door, but officially according to the Dutch law, residents who are living here, patients are our responsibility, so they are not allowed to leave De Hogeweyk without somebody else. Of course, they leave a lot, they go on bus trips, on a walk and a bike tour, etc., but always with somebody else*’.

**Figure 22:**
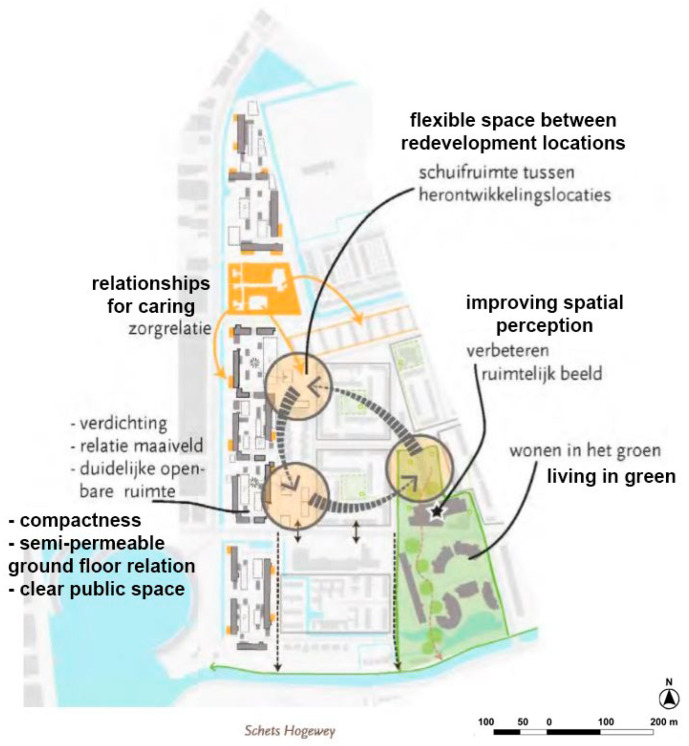
The municipal strategic plan proposes De Hogeweyk as a relevant stakeholder to strengthen relations for care in the immediate surrounding (Weesp Municipality, 2014, p. 92).

The municipal strategy ‘Structuurvisie Weesp 2013–2030’ develops a strategic planning vision that should articulate the future developments of urban actions as well as sectorial urban policies (Figure 22). In this sense, De Hogeweyk is already considered as a special stakeholder in the territory to create new synergies with the surrounding environment.

**Figure 23:**
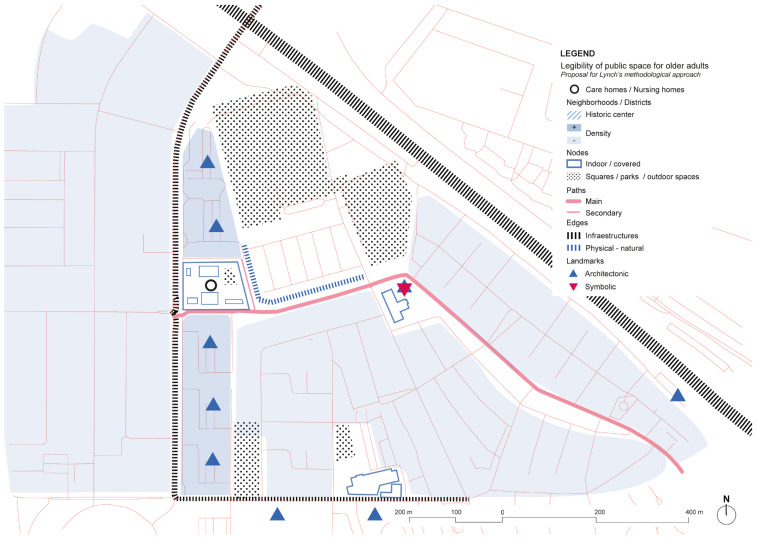
De Hogeweyk surroundings’ scheme based on Lynch’s theory. Source: Own elaboration.

Analysis of the urban surroundings of De Hogeweyk shows a strong zoning area with big sports equipment, large activity hub, and residential developments both in low and high density, creating different tissues in the area. Although nowadays the center is as yet a closed environment, urban strategies aim for developing and strengthening the relationships with De Hogeweyk and the rest of the local settings (Figure 23). The urban environment is similar to those that we find in the outdoor spaces corresponding to the backyards of De Hogeweyk, although from analysis based on Lynch’s theory, several infrastructure barriers are found, which could be difficult pedestrian safety paths.

#### 3.3.2. Kwiek Beweegroute, Nuenen, Eindhoven

**Figure 24:**
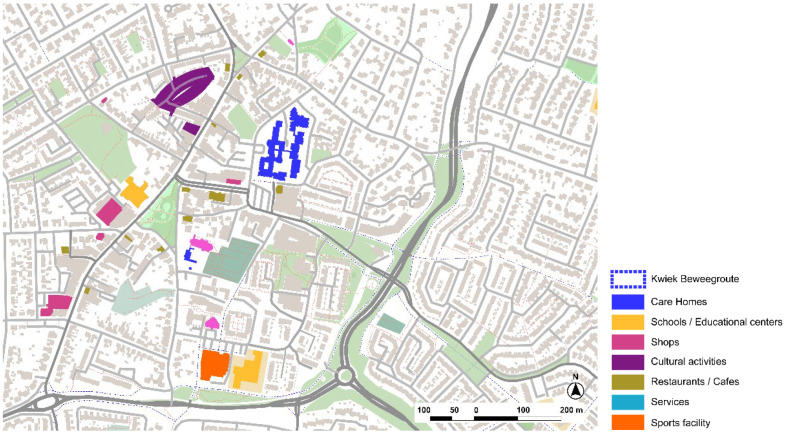
Analysis of the immediate surroundings in which the Kwiek Beweegroute project is set in Nuenen. Source: Own elaboration.

The Kwiek Beweegroute has been implemented in more than 50 cities in Belgium and The Netherlands. Muscular toning, balance, strength, or stretching are some of the exercises that are proposed within the walking path (Figure 24). The project usually starts from a care home or day care facility, as the main purpose is to implement the different programs in the public space so caregivers can easily organize outdoor walks with old people to encourage physical activity and social interaction.

‘*The first question came from the community of Eindhoven to say “OK, we have so many designers in the city, we have some social issues. Let’s define a project. We have in this case, senior neighborhoods where the percentage of seniors is very high”. We had several teams of designers that got into one neighborhood and the subject of the call was public space and seniors. And you had to design a public space not even maybe the most necessary but come with a project that helps society or covers certain issues*’.

**Figure 25:**
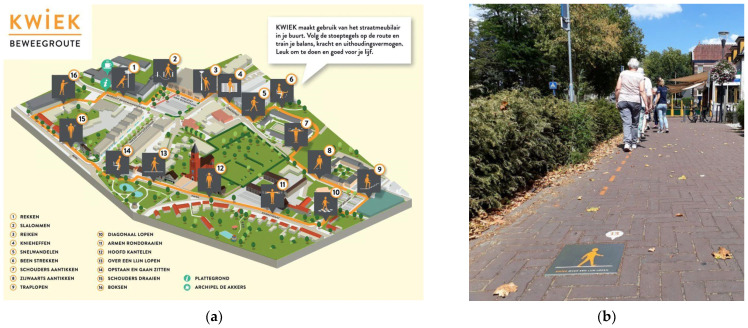
Outline of the Kwiek Beweegroute project. Source: Studio van Laar, Kwiek Beweegroute Nuenen. (**a**) Itinerary with the different exercises across the neighborhood, creating a continuous path for activity and socializing in the outdoor space. (**b**) Micro-elements that are integrated in tiles, urban furniture, and public spaces promote multiple activities and exercises that have been developed with training specialists, physiotherapists, and a design team.

The methodology starts with the analysis of the urban environment and the selection of an accessible pathway in close collaboration with the local municipality team, a hospital or a senior care home. Specific spots are selected to take advantage of pre-existing elements to support the activities. It is also important to connect relevant public spaces for activities to promote social participation and engagement in common urban spaces (Figure 25).

‘*The feeling of being engaged in something constructive and built together that they are part of, and they can use the public space through an interesting itinerary that can be something for them that makes them more inclusive*’.

**Figure 26:**
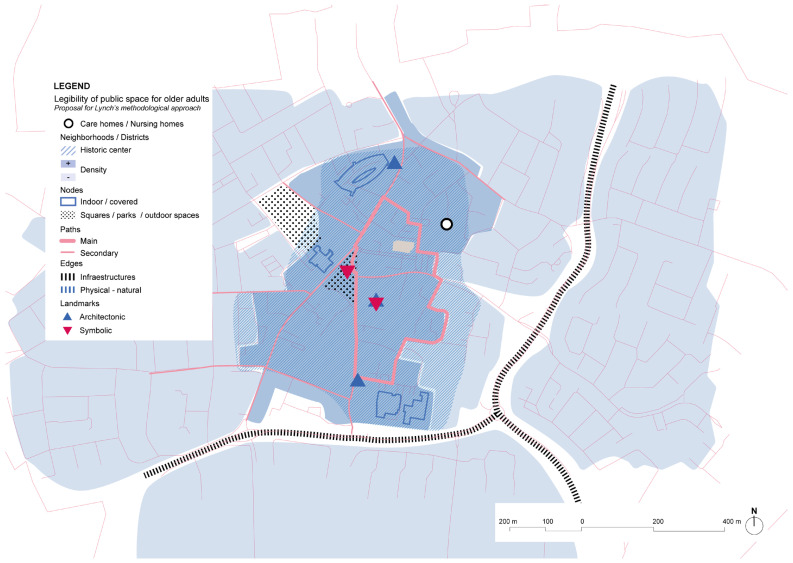
Scheme of the Nuenen’s Kwiek Beweegroute project and its environmental context based on Lynch’s theory. Source: Own elaboration.

The home care center around which the Kwiek Beweegroute has been developed also includes rental apartments for older people. The urban center offers an environment with proximity to a variety of commerce and services. Regarding Lynch’s elements, the main roads of the urban center are kept as soft mobility streets (Figure 26). Barriers are in the periphery due to major infrastructures. On the other hand, pedestrian paths and urban structure might not be clear enough and there is low hierarchy of street types, being sometimes confusing as landmarks are not very prominent. The Kwiek Beweegroute might also offer new elements for wayfinding in the city center.

## 4. Conclusions

The number of people living with dementia is progressively increasing as a result of population ageing and the reduction in fertility rates [27] and this will drive attention to the adaptation of cities as the world’s level of urbanization is expected to increase [28]. However, care systems and solutions are yet unprepared to meet the needs of this trend and the increasing demand of care services by the older population or people with disabilities have become a major priority for governments and institutions in order to find efficient and sustainable solutions for the provision of high quality and person-centered care [29,30]. At the same time, there is a raised interest at the international level to create dementia-friendly societies [1,31], working towards more inclusive environments, both social and physical.

In this context, evidence about dementia-friendly initiatives is usually centered in care settings, usually focusing on environmental design, dementia awareness, and training [5,6]. The lack of evidence has already been acknowledged to evaluate the impact of dementia-friendly communities and their sustainability in long-term planning, as they rely on community networks and volunteers, which makes it unpredictable [32], even though efforts have been made to standardize dementia-friendliness [33].

Regarding the built environment, little evidence has been published regarding outdoor public spaces [34,35,36]. In fact, the literature usually focuses on therapeutic gardens as outdoor spaces [37], whereas the studies that show the impact of the design of the built environment on people with dementia are usually based on sociosanitary environments, either care homes or hospitals [38].

The eight analyzed projects show best practice in different regions that were selected across Europe. In this sense, it is important to highlight the absence of European Mediterranean best-practices in terms of dementia-friendliness in international publications. The study cases mainly show innovations in terms of long-term care homes, where the main strategies that arise are the integration within normalized living, which is translated into a small-scale design in terms of the structure of the care home.

Outdoor spaces play an important role in design, although it varies significantly depending on each context. Outdoor spaces in De Hogeweyk are completely open to people with dementia, as it provides a closed environment to move around safely. It provides a normalized living environment both in the indoor and outdoor spaces, recreating common streets of any other city in The Netherlands. On the other hand, interaction with the local community is, as yet, a target to promote in the coming years. In Belgium, there is a high interest to reduce the stigma and keep people with dementia in relation with their community. The result is outdoor spaces in the immediate surroundings of the care homes that provide intergenerational opportunities to meet and promote social interaction. Finally, the example of United Kingdom also puts the emphasis on integration in the community, but even though they have generous spaces to create outdoor meaningful programs for people with dementia, the urban design of the surroundings is still car-centered.

The rest of the projects that are not care homes put the emphasis on social interactions and accessibility standards, to enable people with dementia to remain engaged in normalized programs and activities that promote both physical and mental health. All the informants acknowledged the importance of adapting the environment to specific necessities of older people with dementia, considering the challenges for safety and security that this can imply.

The analysis of the immediate surroundings, design of public space, and legibility of the urban setting according to Lynch’s theory shows an interesting perspective about how the promotion of the autonomy for people with dementia should consider safe environments, where keeping active, wandering, and continued social interaction in the different stages of the illness are considered. The treatments of streets both in Belgium and in The Netherlands represent good examples of streetscapes where the pedestrian or the soft mobility has the priority. Also, it eliminates the barrier effect in the neighborhoods and helps to be understood as a friendlier environment. On the other hand, low-density residential areas usually lack enough landmarks to support wayfinding and navigation. Some cases incorporate signposts to help people to orient themselves and identify the main facilities of the city. It is important to highlight that the dependency profile of the people with dementia that live or visit the selected study cases varies a lot, depending on the care model and the selected case for study. For example, in De Hogeweyk it is a requirement to have been diagnosed with dementia or Alzheimer’s, while in the WZC De Weister there are also people with high dependency profiles.

This research encountered other important limitations. Firstly, the data that were collected from the study cases were too heterogeneous since the typology of the projects was also diverse and it was difficult to categorize physical elements from the built environment in order to analyze them. Secondly, urban analysis is based on main data from Google Maps and open street maps analysis, as more precision in this sense was out of the scope. Regarding interviews, only one person was the designer, while the rest were directors or in another coordination role. The selection could have included additional criteria to have comparative results from the participation of the subjects; however, in each case, the person that was interviewed was the key informant to explain the project from a wider perspective. Participation of people living in each of the settings to evaluate the perspective of the persons and caregivers would have been the ideal scenario, but it would require a major project to make it possible.

Evidence shows the importance of normalized living environments for people with dementia to support the quality of life by maintaining physical, cognitive, and social functions and promoting the inclusion of the person in the community.

Even though dementia-friendly communities focus on the social inclusion of the person with dementia, case studies show that the physical environment is usually considered as a key facilitator to promote wellbeing. However, dementia-friendly initiatives usually encounter barriers in public space when it is not accessible, or it is difficult to navigate through it. On the other hand, the assumption of some risk is seen as positive when it comes to providing freedom of choice and movement for the person, which reinforces their decision-making process and contributes to improve the wellbeing of the person. Also, the tendency is to create smaller-scale habitats that are integrated into an existing neighborhood in order to keep older people with dementia in their local environment.

In terms of future lines of research, it will be interesting to consider the results of the CASCADE project in United Kingdom, in which Harmonia Village and Harmony House are included. Their results would support the evidence base of the positive impact of such care facilities in the local context. Also, participation of the people with dementia or relatives was not possible for inclusion in this research due to the lack of funding. In this sense, qualitative research on quality of life in the different settings would have been interesting, although many other factors can influence the feeling of wellbeing and it is difficult to establish a direct correlation considering only a short list of elements.

There is a demand for multidisciplinary approaches to better understand the benefits of small-scale projects that are integrated in the community as well as the economic impact. This evidence would support the acceleration of innovation and new opportunities to provide better care services for people with dementia within their communities. However, on the other hand, this qualitative micro-scale analysis should be contrasted with macro-analysis and population trends in order to establish coherent and integrated care strategies in national and international plans.

## Figures and Tables

**Table 1:** Study cases included in the World Alzheimer Report 2020. Source: Own elaboration.

Type of Center	Oceania	Asia	Latin America	North America	Africa	Europe				
						M	N	C	A	O ^1^
Elderly day care center	2	12	2	1	0	1	0	0	3	0
Care home	19	9	1	10	1	2	3	1	7	5
Public equipment	1	1	-	1	-	-	1		1	-
Hospital	-	-	-	-	-	-	-		1	-
TOTAL	22	22	3	12	1	3	4	1	12	5

^1^ Care models in Europe: A = Anglo-Saxon; C = Continental; N = Nordic; M = Mediterranean; O = Others (Croatia, Estonia, Poland and Switzerland).

**Table 2:** List of the selected study cases. Source: Own elaboration.

City	Type of Project	Name and Details	Criteria
*1. United Kingdom*			
1.1. Newcastle-upon-Tyne	Dementia-friendly city	Discovery museum	International reference on Active Ageing: National Centre of Active Ageing (NICA) and community local initiatives
1.2. Dover	Dementia village	Harmonia Village, new residential care concept for people with dementia	European project CASCADE selected in competitive call, founded by European Union, Interreg 2Seas Mers Zeeën Program (2017–2023)
1.3. Rochester	Respite Center	Community center with different services for people with dementia and relatives	
*2. Belgium*			
2.1. Kortrijk	Reminiscence walks	Care home WZC de Weister	European Award EFID 2014
2.2. Bruges	Dementia-friendly Bruges	FOTON. Regional Expertise Centre. Familierzorg West-Vlaanderen VZW (Family Care in West-Flanders)	European Award EFID 2012 and 2020 ‘*Together for a dementia-friendly Bruges*’. Golden Ear for Foton Volunteers
2.3. Osterloo, Geel	Small-scale care home	Huis Perrekes	Best practice nomination in ADI, 2017. Presentation at ‘*International Mind Conference 2019*’. Publication in Flanders Architectural Review No. 14. ‘*When Attitudes Take Form*’.
*3. The Netherlands*			
3.1. Weesp	The Hogeweyk, dementia village	Care home for people living with dementia.	Several publications at national and international level. Awards and prizes: Hospitality Care Award 2010 Nominated for the Hedy d’Ancona Award 2010, excellence in Healthcare architecture Golden label for Quality by Perspect 2006 Project of the World, Expo 2000 Hannover International Hospital Federation Award 1995 Dien Cornelissenprijs 1993
3.2. Nuenen	Active walks: Kwiek beweegroute	Healthy and activity walks in local neighbors around care homes.	First prize UKON Uniek Prijs 2014 Third prize Your Street Design Indaba 2012

**Table 3:** List of questions for interviews. Source: Own elaboration.

*Interviews’ Script*
1. How many people live in the community? How many people regularly visit the community? What is the level of dementia and dependency?
2. What is the average number of years a person spends living in the community?
3. Do you know how many people (without dementia) visit and join the community? What profile? What kind of activities do they join?
4. Is there any relationship with other institutions that support and collaborate with the care home? (schools, universities, healthcare services…)
5. How many volunteers participate normally? What kinds of activities are organized?
6. Is the care home well connected to the city center by public transport?
7. What kind of buildings or services are next to the care home? Is there any relationship (use/function/activities…)?
8. Is the care home designed considering any of the dementia-friendly guidelines? Which ones? Which outdoor spaces do residents usually use?
9. Is there any special consideration about housing/public spaces/transportation/design guidelines in a local Urban Master Plan?
10. Is design conceived from the dementia-friendly guidelines? How?
11. How can design be adapted to create more inclusive environments both indoor and outdoor from your experience?
12. Is there any project or planning about the proximity of green areas? Therapeutic gardens/horticultural activities?
13. How do you work with sensorial activities and design?
14. Is the public space accessible enough to promote autonomy? Do people with dementia use it? Existence of benches, outdoor features, iconic landmarks, and buildings, etc.
15. Are features such as thermal/lighting/wind comfort considered in the design?
16. In your opinion, what is the most important thing of your community for people with dementia? What are the main initiatives that are being developed in your city/community for the dementia-friendly community?
17. What features in public space do you think are the most valuable for people with dementia? Is there any specific built environment in the city (square, market, garden, healthcare residence…) that is adapted and how for people with dementia?

**Table 4:** List of interviews. Source: Own elaboration.

Project	Charge/Role	Duration [Min]
*1. United Kingdom*		
1.1. Discovery Museum, Dementia-friendly Newcastle-upon-Tyne	Leader and champion of the Platinum Program	40′
1.2. Harmonia Village, Dementia village, Dover	Director of the new care home Harmonia Village	**38′**
1.3. Harmony House, Respite Center, Rochester	Director of the new respite center	**83′**
*2.Belgium*		
2.1. WZC De Weister, small-scale care home, Aalbeke, Kortrijk	Director of 2 care homes in Kortrijk, one of which is WZC De Weister	112′
2.2. Fotonhouse, Dementia Expertise Centre, Bruges	Director of the Familiezorg West-Vlaanderen VZW and coordinator of the project Foton	23′
2.3. Huis Perrekes, Geel, Osterloo	Worker at Huis Perrekes	84′
*3. The Netherlands*		
3.1. De Hogeweyk, Weesp	CEO and senior consultant, co-founder of De Hogeweyk	166′
3.2. Kwiek Beweegroute, Nuenen	Founder of the Kwiek beweegroute	24′

## Data Availability

Not applicable.
